# Anti-Oxidant and Anti-Inflammatory Substance Generated Newly in Paeoniae Radix Alba Extract Fermented with Plant-Derived *Lactobacillus brevis* 174A

**DOI:** 10.3390/antiox10071071

**Published:** 2021-07-02

**Authors:** Shrijana Shakya, Narandalai Danshiitsoodol, Sachiko Sugimoto, Masafumi Noda, Masanori Sugiyama

**Affiliations:** 1 Department of Probiotic Science for Preventive Medicine, Graduate School of Biomedical and Health Sciences, Hiroshima University, Hiroshima 734-8551, Japan; d206672@hiroshima-u.ac.jp (S.S.); naraa@hiroshima-u.ac.jp (N.D.); bel@hiroshima-u.ac.jp (M.N.); 2 Department of Pharmacognosy, Graduate School of Biomedical and Health Sciences, Hiroshima University, Hiroshima 734-8551, Japan; ssugimoto@hiroshima-u.ac.jp

**Keywords:** *Lactobacillus brevis*, Paeonia Radix, pyrogallol, antioxidant, anti-inflammatory, RAW 264.7 macrophage cells

## Abstract

Fermentation of medicinal herbs can be a significant technique to obtain bioactive compounds. Paeoniae Radix (PR) used in the present study is a well-known herbal medicine that exhibits anti-inflammatory and immunomodulatory activity. The aim of this study is to explore the possibility that a bioactive compound is newly generated in PR extract by fermentation with a plant-derived lactic acid bacteria *Lactobacillus brevis* 174A. We determined the anti-inflammatory activities in lipopolysaccharide (LPS)-stimulated RAW 264.7 macrophage cells. The PR extract fermented with *Lactobacillus brevis* 174A markedly increased the total phenolic content, decreased intracellular ROS levels, inhibited the release of nitric oxide (NO). It also suppressed inflammatory cytokines IL-6, TNF-ɑ, while simultaneously downregulating the gene expressions of iNOS, IL-6, TNF-ɑ, and IL-1β compared to the unfermented PR extract. Furthermore, the bioactive compound newly generated from the fermentation was identified as pyrogallol. It inhibits the inflammatory responses in a dose-dependent manner suggesting that fermentation of the herbal extract used as a medium together with the plant-derived lactic acid bacterial strain may be a practical strategy to produce medicines and supplements for healthcare.

## 1. Introduction

Fermentation technology, which has been developed as an indispensable method of food preservation, has also contributed to the manufacture of functional foods. Among different microorganisms, lactic acid bacteria (LAB) are mostly used for industrial fermentation due to numerous potential health effects [1]. LAB can be derived from numerous sources [2]. On the basis of isolated environment, it can be roughly classified into animal derived and plant-derived LAB. The animal-derived LAB strains are isolated from the skin, gastrointestinal and urogenital tracts of humans and animals, and have been widely used in yogurt and cheese preparation. The plant-derived ones, isolated from various plant sources, have been useful to produce fermented foods, mainly in East and Southeast Asia [3]. The plant-derived LAB strains are much more resistant to artificial gastric juices and bile than animal-derived ones [4]. Our research group has isolated over 1000 strains of plant-derived LAB strains from fruits, vegetables, flowers, and medicinal plants. Several of the LAB strains have demonstrated health benefits like immune modulation, the improvement of liver function and reduction of obesity [5,6,7,8]. Furthermore, the plant-derived LAB strains could convert some compounds contained in medicinal herbs to bioactive substances [9].

Plants, as the inherent reservoirs of bioactive compounds like glycosides, antioxidants, phenolics, and dietary fibers, as well as vitamins and minerals, enable LAB to follow various metabolic routes, depending on species and strains [10]. Thus, the plant fermentation process by LAB causes decomposition and/or biotransformation of complex substrates into compatible components. This can improve the bioavailability and bioactivity of phytochemicals, as well as cause a marked increase of functional microbial metabolites with beneficial consequences for human health [11]. In particular, bacterial conversions of glycosides, phenolic acids, and tannins to biologically active metabolites through glycosyl hydrolase, phenolic acid decarboxylase and reductase, and esterase activities during plant fermentation have garnered much interest [12].

The dried root of *Paeonia lactiflora* Pall, also known as Paeoniae Radix Alba (PR), has been used for centuries as a medicinal herb in Japan, China, and Korea for the treatment of rheumatoid arthritis, systemic lupus erythematosus, hepatitis, dysmenorrhea, muscle cramping, and spasms, as well as fever for centuries [13]. Its water/ethanol extract is referred to as total glucosides of peony (TGP), which contains more than 15 components including paeoniflorin, oxypaeoniflorin, albiflorin, benzoyl albiflorin, gallic acid, pentagalloylglucose, paeonol, and benzoic acid [14]. Paeoniflorin, which is the most abundant compound, is responsible for the in vivo as well as in vitro activities. However, it is also reported to have poor oral absorption and low bioavailability due to extensive metabolism by the intestinal microbiota. Hence, some bioactivities of TGP have been associated with bioactive gut microbial metabolites including benzoic acid [15,16,17].

It has been found that the fermentation of *Artemisia princeps* Pampanini herb extract with plant-derived *Lactobacillus (Lb.) plantarum* SN13T generates catechol and seco-tanapartholide C as IL-8 release inhibitors [9]. In this study, we fermented PR extract with *Lb. brevis* strain 174A isolated from *Citrus iyo* [18], and compared its anti-oxidant and anti-inflammatory bioactivity to that of PR extract without fermentation in lipopolysaccharide stimulated RAW 264.7 macrophage cells. Furthermore, we identified a bioactive compound newly produced after fermentation as pyrogallol.

## 2. Materials and Methods

### 2.1. Bacteria Culture and Fermentation Conditions

*Lb. brevis* 174A, which has been isolated from a *Citrus iyo*, was grown at 37 °C overnight in MRS broth (Merck, Germany). After the cultivation, the bacterial cells were collected by centrifugation and resuspended with sterile 0.85% (*w/v*) NaCl solution.

Paeonia Radix Alba (5 g), purchased from Kojima Kampo Co., Ltd. (Osaka, Japan), was suspended in 100 mL of distilled water and boiled for 30 min. After cooling to room temperature, the PR extract was obtained by centrifugation at 5000× *g* for 10 min, filtrated with a 0.22 µm membrane filter (Advantec Toyo Co., Ltd., Tokyo, Japan). Then, the 174A cells suspended in the sterile 0.8% (*w/v*) NaCl solution were inoculated at a final 1% *v/v* in the PR extract and incubated for the given times (24, 48, 72, and 96 h) at 37 °C. For unfermented extracts, only sterile 0.85% (*w/v*) NaCl solution was used. The extracts were finally collected after centrifugation at 5000× *g* for 10 min and followed by filtration.

### 2.2. Measurement of Total Phenolic Content (TPC)

The total phenolic content (TPC) in the fermented and unfermented extracts was determined by the Folin–Ciocalteu colorimetric method. To 10 µL of each extract, 100 µL of 10% Folin–Ciocalteu reagent was added. After 3 min, 90 µL of sodium carbonate (1N) solution was added and incubated for 1 h. The absorbance was measured at 765 nm, and the TPC was expressed as µg/mL of gallic acid equivalent (GAE). Experiments were analyzed at least three times and with triplicate samples.

### 2.3. Cell Culture and Treatment

The RAW 264.7 macrophage cells were grown in DMEM medium supplemented with 10% FBS and 100 µg/mL of penicillin/streptomycin. The cells were incubated in a humidified 5% CO_2_ atmosphere at 37 °C. To stimulate the cells, the medium was exchanged with fresh DMEM medium, and 1 µg/mL LPS was added in the presence or absence of the extracts (1% (*v/v*) final concentration) or pyrogallol (FUJIFILM Wako Pure Chemical Co., Osaka, Japan) solution (1 or 5 µg/mL) and incubated for 6 or 24 h.

### 2.4. Cell Viability Assay

Cytotoxicity was analyzed using a CCK-8 kit (Dojindo Laboratories, Kumamoto, Japan) according to the manufacturer’s instructions. Briefly, 100 μL of cells at a density of 1.8 × 10^5^ cells per ml was incubated with LPS (1 µg/mL) in the presence or absence of the extracts or pyrogallol for 24 h. CCK-8 solution (10 μL) was added to each well, and the cells were incubated for another 1 h. Then, the absorbance at 450 nm was measured. The percentage of cell viability was determined as a value relative to untreated cells.

### 2.5. Measurement of Intracellular ROS Levels

The RAW 264.7 macrophage cells were treated with LPS in the presence or absence of the extracts or pyrogallol for 24 h. The cells then were incubated with 10 mM DCFH-DA for 30 min at 37 °C and washed twice with phosphate-buffered saline. Subsequently, the DCF fluorescence was measured at excitation and emission wavelengths of 485 and 530 nm, respectively [19].

### 2.6. Measurement of NO Production

The productivity of NO was evaluated by measuring the nitrite in the supernatant from the cell culture treated with LPS for 24 h. Equal volumes of the supernatants and Griess reagent (1% sulfanilamide in 5% phosphoric acid and 0.2% naphthyl ethylenediamine dihydrochloride) were mixed and incubated at room temperature for 5 min. The absorbance at 550 nm was measured, then the NO concentration was calculated from the standard curve of sodium nitrite [20].

### 2.7. Inflammatory Cytokine Determination

The cells (1.8 × 10^5^ cells per ml density in 24 well plate) were treated with LPS in the presence or absence of the extracts or pyrogallol for 24 h. The concentrations of inflammatory cytokines IL-6 and TNF-α in the cell supernatants were determined by respective ELISA kits (BioLegend, San Diego, CA, USA).

### 2.8. RNA Extraction and qRT-PCR Analysis

The total RNA from cells treated for 6 h with fermented or unfermented extracts were isolated using NucleoSpin RNA plus (Macherey-Nagel GmbH & Co. KG, Duren, Germany), and reverse-transcribed using the ReverTra Ace qPCR RT master mix with gDNA remover (Toyobo, Japan) according to each manufacturer’s instruction manual. The qRT-PCR was conducted on the PikoReal real-time PCR system (Thermo Fisher Scientific, Inc., Waltham, MA, USA) using the KAPA SYBR Fast qPCR Kit (Kapa Biosystems, Wobum, MA, USA). The qPCR was carried out under the following conditions: an initial 2 min at 95 °C, followed by 38 cycles of 5 s at 95 °C and 10 s at 60 °C. The relative transcriptional levels, which were normalized to a housekeeping gene (GAPDH). The gene expressions of IL-6, TNF-α, IL-1β and iNOS were analyzed using the ΔΔCT method. The primers used in this experiment are as follows: 5′-GACATCATACTTGGCAGG-3′ and 5′-CTCGTGGAGTCTACTGGT-3′ for GAPDH; 5′- ACAGGTCTGTTGGGAGTGGTATC-3′ and 5′-CTCTCTGCAAGAGACTTCCATCC-3′ for IL-6; 5′-AGCCCCCAGTCTGTATCCTT-3′ and 5′-CTCCCTTTGCAGAACTCAGG-3′ for TNF-α; 5′-ATGGCAACTGTTCCTGAACTCAACT-3′ and 5′-CAGGACAGGTATAGATTCTTTCCTTT-3′ for IL-1β; and 5′-GGTGTTGAAGGGGTAGCTGA-3′ and 5′-ATCATGGACCACCACACAGC-3′ for iNOS [12].

### 2.9. HPLC Analysis, Extraction, and Identification of a Newly Produced Compound in the Fermented Extract

A 2.5 µL aliquot of the extracts applied to HPLC (JASCO system; JASCO Corporation) with Triart Bio C4 (5 µm, 30 nm, Φ = 3 mm, L = 150 cm) column (YMC Co., Ltd., Kyoto, Japan) was eluted with 15% acetonitrile for 8 min at a flow rate of 0.5 mL/min. The elution profile was monitored at an absorbance of 220 nm. The chromatogram of the fermented extract was compared with that of unfermented extract to observe the peaks of compounds newly produced by the fermentation.

For extraction of the supposed compound, we used a TLC guided organic solvent extraction method. Briefly, the PR extract, fermented for 48 h, was extracted three times with an equal volume of ethyl acetate, dried, and 10 times concentrated in ethyl acetate. Further, it was mixed with an equal volume of hexane, and the upper layer was dried and dissolved in water, which was further extracted with chloroform ^1^H-NMR and ^13^C-NMR spectra of the isolated compound was taken on a JEOL JNM-LA500 spectrometer at 500 and 125 MHz, respectively. MS spectra were taken on Thermo Fisher Scientific LTQ Orbitrap XL (HR-ESI-MS) at the Natural Science Center for Basic Research and Development (N-BARD), Hiroshima University.

Spectroscopic data of pyrogallol: ^1^H-NMR (Methanol-d_4_, 500 MHz): 6.29 (2H, d-like, J = 8.0 Hz), 6.47 (1H, t-like, J = 8.0 Hz); ^13^C-NMR (Methanol- d_4_, 125 MHz): 108.5, 120.3, 134.5, 147.4; HR-ESI-MS (Negative-Ion Mode) m/z: 125.0243 [M-H]- (Calculated for C_6_H_5_O_3_: 125.0244).

### 2.10. Statistical Analyses

All measurements were performed in triplicate, but the fermentation experiments were done in duplicate. Data were presented as means and SD of replicates. Statistical analyses were performed with SPSS version 20. The significance of differences was determined via ANOVA, followed by a post hoc Tukey test, and differences with *p* < 0.05 were considered statistically significant.

## 3. Results and Discussion

### 3.1. Simultaneous Increase in Total Phenolic Content and Bioactivity in the Fermented PR

**Figure 1:**
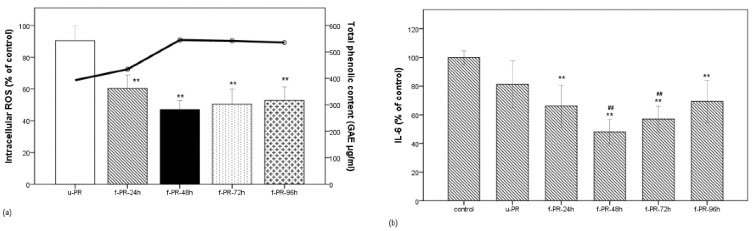
Correlation between phenolic compound content and bioactivity of the PR extracts fermented for the given times. (**a**) Correlation between phenolic content (line) and the intracellular ROS level (bar) in the unfermented PR extract and the PR extract fermented for 24, 72, or 96 h in the LPS-stimulated RAW 264.7 macrophage cells; (**b**) IL-6 level (in % of control) under the same condition as (**a**). Data are expressed as the mean value of at least triplicate experiments. Error bars represent +/− standard deviation. ** *p* < 0.01 versus control, ## *p* < 0.01 versus u-PR. u-PR: PR extract without fermentation; f-PR-24h, f-PR-48h, f-PR-72h, and f-PR-96h: PR extract after fermentation for 24, 48, 72, and 96 h, respectively.

In the present study, when the water extract of PR was fermented with Lb. brevis 174A, the TPC in the extract, measured in gallic acid equivalent, changed time-dependently as shown in Figure 1a. We found that the highest amount, 545.05 µg/mL GAE, was reached within 48 h of incubation. This was significantly higher than the amount during the initial 24 h of fermentation and that without fermentation; however, the slight decline in TPC after 48 h was not found to be significant. Apart from monoterpenoid glycosides, the PR extract is reported to be rich in tannins and other phenolic compounds in the form of pentagalloylglucose, gallic acid, methyl gallate, and paeonol [14]. Although the changes in phenolic compounds might be due to the enzymatic actions of any of the commonly inherited enzymes of Lactobacillus species, such as glucosidase, esterase, or phenolic acid decarboxylase [21], or even due to the more specifically possessed enzyme tannase [22], here with regard to the known constituents of the PR extract, the role of a decarboxylase seems more likely. Moreover, there are numerous reports demonstrating changes in TPC associated with simultaneous changes in antioxidant activities after Lactobacillus fermentation of various food products, among which a majority have reported simultaneous increases [23,24,25,26].

The bioactivity of medicinal herbal extracts on inflammatory and oxidative responses was evaluated by using the RAW264.7 murine macrophages stimulated with LPS [20]. LPS binds to Toll-like receptor 4 (TLR-4) of the immune cells such as macrophages, followed by the activation of the signaling pathways such as NF-κB. Subsequently, proinflammatory cytokines including IL-1β, TNF-α, and IL-6 are liberated, followed by the generation of NO and ROS. Although their ultimate purpose is to provide localization on the infected cells, remove the causative agent, and facilitate a healing process through their chemotactic and vasoactive properties, detrimental chronic inflammation occurs when their levels are elevated for a prolonged period of time [27]. Therefore, anti-inflammatory compounds that can downregulate these mediators can be beneficial in inflammatory disorders. In this study, RAW264.7 cells were stimulated with LPS (1 µg/mL) for 6–24 h, which was observed to be adequate to induce significant levels of IL-6, TNF-α, and NO levels in the cell supernatant, increase the intracellular ROS, as well as significantly increase the gene expressions of IL-1β, IL-6, TNF-α, and iNOS (inducible NO synthase) (data shown in Supplementary Figure S1a,b).

As shown in Figure 1a, when the LPS-stimulated cells were treated with the unfermented extract (u-PR) or the extract (f-PR) fermented for the given times (24–96 h each: f-PR-24h, f-PR-48h, f-PR-72h, and f-PR-96h), the intracellular ROS level was decreased in all groups treated with fermented extracts as compared to that treated with the unfermented one. The highest decrease was observed in the f-PR-48h extract. Figure 1b shows that the IL-6 levels in extracts with and without fermentation were not decreased. It shows that the IL-6 levels released in all of the treated cells were lower than in the untreated cells, i.e., <100%, and significantly lower than in the cells treated with the fermented extracts, when compared to those treated with the u-PR extract. Interestingly, the IL-6 level was also the lowest (less than 50% of the control) in the f-PR-48hr-treated cells, indicating that the higher bioactivities correspond to the higher TPC of the fermented extract in f-PR-48h.

### 3.2. Significant Decrease in Inflammatory Gene Expression and Release of NO and TNF-α by Fermented PR Extract

**Figure 2:**
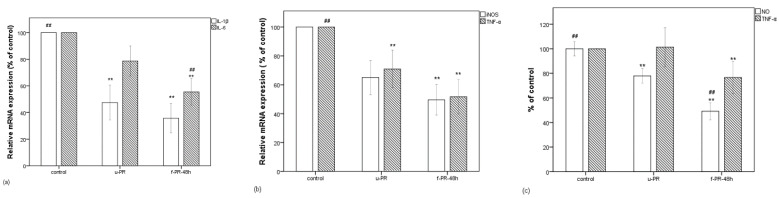
(**a**) Decrease in the mRNA expressions of IL-6 and IL-1β (in % of control) in LPS-stimulated RAW 264.7 cells, which were treated with unfermented PR extract and the PR extract fermented for 48 h. (**b**) Decrease in the mRNA expressions of iNOS and TNF-α (in % of control). (**c**) Decrease in NO and TNF-α levels (in % of control). Data are expressed as the mean value of at least triplicate experiments. Error bars represent +/− standard deviation. ** *p* < 0.01 versus control, ## *p* < 0.01 versus u-PR. u-PR: PR extract without fermentation; f-PR-48h: PR extract fermented for 48 h.

The relative gene expression levels of IL-1β, IL-6, iNOS, and TNF-α in the RAW 264.7 cells stimulated with LPS for 6 h, were determined in the presence of the u-PR or f-PR-48h. As shown in Figure 2a,b, the expressions of IL-1β and TNF-α in the RAW 264.7 cells treated with either u-PR or f-PR-48h were significantly decreased. However, that of iNOS was significantly decreased only by the f-PR-48h treatment. In addition, the IL-6 expression was also lowered by f-PR-48h than by the u-PR treatment. Further, we also measured the release levels of nitric oxide (NO) and TNF-α released as inflammatory mediators in the supernatant from the LPS-stimulated RAW 264.7 24 h macrophages. As shown in Figure 2c, the release of NO was significantly reduced by either u-PR-treated or f-PR-48hr-treated cells, that of TNF-α was significantly lower in the cells treated with f-PR-48hr when compared to the u-PR-treated cells, demonstrating that the PR extract fermented for 48 h with Lb. brevis 174A inhibits the release of the inflammatory mediators.

The water/ethanol extract of Paeonia Radix has been reported to suppress the production of inflammatory mediators such as prostaglandin E2, leukotriene B4, and NO, decrease intracellular Ca^2+^, and inhibit oxidative stress to mediate anti-inflammatory effect in animal models [13]. Paeoniflorin, one of major constituent of Paeonia, was shown to protect the growth of RAW 264.7 macrophage cells from LPS-induced toxicity, decrease the NO and PGE_2_, and repair DNA damage [28].

As shown in a previous report, the ethanol extract of the peony root, containing gallic acid and methyl gallate, plays a role as a free radical scavenger in 2,2-diphenyl-1-picryl hydrazyl (DPPH) radical generation and has inhibitory effect on the lipid peroxidation without any pro-oxidant effect [14]. Our present study demonstrates that the u-PR extract significantly decreased some LPS-induced inflammatory responses in RAW 264.7 macrophages; this ability was then markedly increased after fermentation with *Lb. brevis* 174A. Particularly, the 48 h fermented extract had the highest activity, which could be correlated with the simultaneous change in TPC. As mentioned above, the increase in TPC in the fermented extracts could be associated with the increase in antioxidant activity leading to the significant decrease in intracellular ROS levels. In addition, the correlation of this increase in TPC with the downregulation of pro-inflammatory genes is noteworthy. Therefore, we speculated that some phenolic metabolites having antioxidant and anti-inflammatory properties are newly produced after the *Lb. brevis* 174A fermentation of the PR extract.

### 3.3. HPLC Analysis and Identification of a Newly Produced Compound in the Fermented Extract

**Figure 3:**
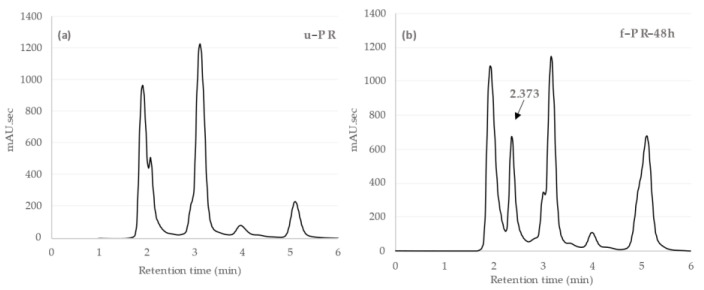
(**a**) HPLC chromatogram of PR extract without fermentation (u-PR) and (**b**) HPLC chromatogram of PR extract after 48 h fermentation (f-PR-48h). A new peak appeared at the retention time (Rt) 2.373 min, indicated by an arrow.

We compared the HPLC analysis of the unfermented PR extract with that of the extract fermented with *Lb. brevis* 174A for 48 h, as shown in Figure 3. There was a new peak at the retention time of 2.37 min in the fermented extract. Similar to the TPC changes, we observed time-dependent changes in the peak area of this compound, with the highest area at 48 h of fermentation. This result implied that the compound, undetected in the unfermented extract and appearing only after fermentation with *Lb. brevis* 174A, could be the supposed bioactive phenolic metabolite contributing to the observed significant changes in the biological properties of the PR extract. Previous studies have also reported phenolic metabolism being abundant to different plant-associated LAB, including *Lb. brevis*, and is hypothesized that it plays a role in preserving energy balance [12]. Different phenolic metabolites, such as catechol, protocatechuic acid, p-hydroxy benzoic acid, gallic acid, caffeic acid, can be produced by different *Lactobacillus* species [12]. Specifically, *Lb. brevis* strains were found to be involved in gallic acid metabolism in green tea and black tea extracts and kaempferol production in cactus cladodes [29,30].

Consequently, the supposed compound was extracted from the fermented PR extract using three equal volumes of ethyl acetate. After mixing the concentrated ethyl acetate fraction with an equal volume of hexane, the upper layer was dried and further extracted with chloroform to get the isolated compound. To determine the chemical structure, ^1^H-NMR and ^13^C-NMR, and ESI-MS were used. The MS spectrum of the compound showed an ion at m/z 125.024, suggesting that its molecular formula is C_6_H_6_O_3_. As a result, the compound was identified as pyrogallol. The NMR spectrum of the isolated sample of pyrogallol is included in the Supplementary Figure S2.

**Figure 4:**
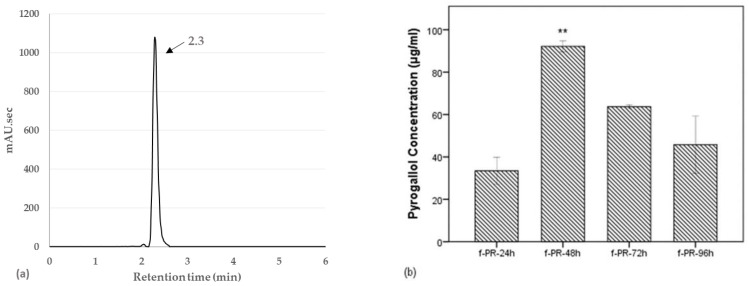
(**a**) HPLC chromatogram of pyrogallol. (**b**) Time-dependent changes in the pyrogallol concentration in PR extracts fermented for 24–96 h. The highest concentration at 92.138 µg/mL was observed in f-PR-48h. Data are expressed as the mean value of duplicate experiments. Error bars represent +/− standard deviation. ** *p* < 0.01 versus f-PR-24h. f-PR-24h, f-PR-48h, f-PR-72h, and f-PR-96h: PR extract after fermentation for 24, 48, 72, and 96 h, respectively.

As shown in Figure 4a, we obtained the chromatogram of standard pyrogallol with its peak at retention time of 2.3 min. A new peak, which was observed at 2.37 min in the previous chromatogram of f-PR-48h, is clarified to be pyrogallol. We also determined the time-dependent concentration of pyrogallol in the PR extracts fermented for 24–96 h. As shown in Figure 4b, the highest concentration (92.138 µg/mL) of the pyrogallol was observed with fermentation for 48 h, which in turn indicates that the observed bioactivity after the fermentation was caused by the pyrogallol. Several studies have reported that the constituents of PR are extensively metabolized by the intestinal microbiota; however, those studies have been focused mainly on the monoterpenoid glycosides. Paeoniflorin, as well as oxypaeoniflorin and benzoylpaeoniflorin, is known to be converted into three metabolites (paeonimetaboline I, II, and III) by intestinal bacteria [17]. Paeoniflorin was also converted to new derivatives of paeonimetabolin I in the presence of various sulfhydryl compounds by *Lb. brevis* [15]. Another study demonstrated that the metabolic pathways of paeoniflorin in human intestinal microflora contained extensive metabolic reactions such as hydrolysis, oxidization, and conjugation [31]. In a recent study, benzoic acid produced via carboxylesterase-mediated gut microbiota decomposition was identified as the bioactive metabolite that mediated the anti-depressant action of paeoniflorin and albiflorin in the central nervous system [16,32].

Pyrogallol is a simple phenolic compound, that can be produced from gallic acid by microbial gallate decarboxylase action [33]. The enzyme-encoding gene has been found to be possessed by several lactic acid bacteria, including *Lb. brevis* [22]. It has been reported that pyrogallol is found in Awaban tea fermented with some *Lactobacillus* spp. Fermentation of the tea increases its phenol content and anti-glycation activity [34]. Moreover, it was demonstrated that green and black teas fermented with *Lb. brevis* 145 were found to contain pyrogallol generated from gallic acid. The LAB strain hydrolyzes the ester bond linked with galloyl groups causing a marked reduction in gallic acid and subsequent production of pyrogallol. This led to the enhanced antioxidant activity and cellular uptake of phenolic compounds following in vitro digestion [30]. It is highly possible that part of the gallic acid freed from gallic acid derivatives was further converted to pyrogallol in our study. *Proteobacteria* and *Firmicutes* bacteria in the human intestinal flora have been demonstrated to harbor gallate decarboxylase [35]. Bearing resemblance to fermentation processes, pyrogallol is formed as a major absorbable dietary gallotannin metabolite in human intestine, an observation that could be of considerable relevance to the in vivo significance of pyrogallol [36].

### 3.4. Pyrogallol as a Sole Bioactive Compound

**Figure 5:**
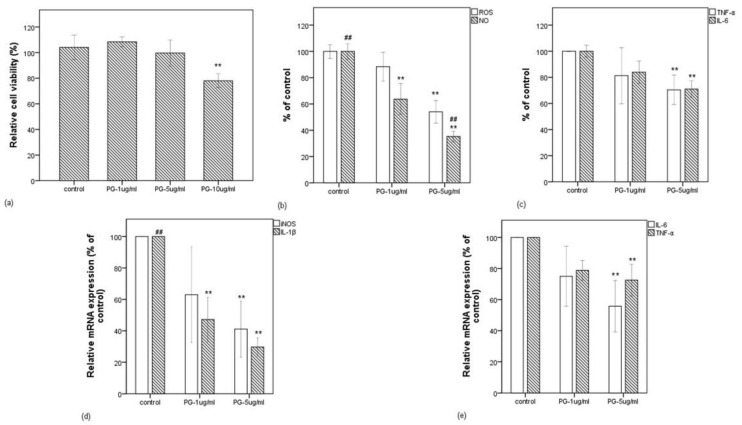
Effect of pyrogallol on LPS-induced RAW 264.7 cells. (**a**) Relative cell viability when RAW 264.7 cells were treated with LPS in the presence of pyrogallol at 1, 5, and 10 µg/mL. (**b**) Effect of pyrogallol at 1 and 5 µg/mL in intracellular ROS and NO release levels (in % of control) in LPS-stimulated RAW 264.7 cells. (**c**) Effect of pyrogallol at 1 and 5 µg/mL in IL-6 and TNF-α release levels (in % of control) in LPS-stimulated RAW 264.7 cells. (**d**) Effect of pyrogallol at 1 and 5 µg/mL in mRNA expressions of iNOS and IL-1β (in % of control) in LPS-stimulated RAW 264.7 cells. (**e**) Effect of pyrogallol at 1 and 5 µg/mL in mRNA expressions of IL-6 and TNF-α (in % of control) in LPS-stimulated RAW 264.7 cells. Data are expressed as the mean value of at least triplicate experiments. Error bars represent +/− standard deviation. ** *p* < 0.01 versus control, ## *p* < 0.01 versus PG-1 µg/mL. PG: pyrogallol.

As described above, 92.138 (±2.556) µg/mL concentration of pyrogallol was determined in f-PR-48h. Thus, corresponding to the 1% (*v/v*) final concentration of f-PR-48h, we evaluated the effect of pyrogallol in the final concentration range (1–10 µg/mL) on the viability of the macrophage cells stimulated with LPS. As shown in Figure 5a, pyrogallol at 1 and 5 µg/mL did not decrease the cell viability, but obviously decreased it at 10 µg/mL. We have chosen the range of 1–5 µg of pyrogallol/mL for further experiments. This range of concentration is within a physiological range, which can be expected to occur in vivo upon metabolism of food or dietary supplement [36].

Figure 5b,c shows that the LPS-induced intracellular ROS was significantly decreased by pyrogallol at 5 µg/mL and was followed by the release of IL-6 and TNF-α, but NO was decreased even at 1 µg pyrogallol/mL, suggesting that pyrogallol functions in a concentration-dependent manner on the inflammatory mediators. This possibility was clarified by the qRT-PCR method. As shown in Figure 5d,e, although pyrogallol inhibited the expressions of IL-1β, iNOS, IL-6, and TNF-α at 5 µg/mL, that of IL-1β was inhibited strongly even at 1 µg/mL. Noteworthily, the bioactivity expressed by the extract fermented for 48 h is comparable to that expressed by pyrogallol at 5 µg/mL.

The present study indicates that pyrogallol solely decreases the LPS-stimulated inflammatory responses in a concentration-dependent manner. Consistent with that finding, pyrogallol was previously identified as the active compound in an *Emblica officinalis* extract that regulated the expression of pro-inflammatory genes in bronchial epithelial cells [37]. Further, it has been revealed that pyrogallol (5 mg/L) can cause a microbial metabolite inflammation in the RAW 264.7 macrophage cells through the activation of the AMPK pathway and the suppression of NF-kB activity [38]. Pyrogallol was also identified in *Rodgersia podophylla* leaves as an inducer of the Nrf2/HO-1 pathway and an inhibitor of NF-κB and the MAPKs pathway in RAW 264.7 cells [39]. The catechin metabolites and pyrogallol detected in post-fermented teas were reported to have an anti-allergic effect that suppresses nasal symptoms and IL-9 gene expression [40]. Pyrogallol has also been demonstrated to function as an anti-carcinogenic [36] and anti-proliferative [41] compound and as an inhibitor of xanthine oxidase [42].

## 4. Conclusions

This study demonstrates that the medicinal herb Paeonia Radix Alba extract fermented with the plant-derived *Lb. brevis* 174A increases its total phenolic content. It is associated with increased intracellular antioxidant effect, decreased release of inflammatory mediators, as well as the downregulation of pro-inflammatory genes in activated macrophages. In this study, pyrogallol, which is a metabolite of gallic acid, was identified as a new compound that was generated in the herbal extract fermented with *Lb. brevis* 174A. We conclude that medicinal herbal extract fermented with the plant-derived LAB strains could be a significant technique for enhancing their therapeutic potential.

## Data Availability

All data sets generated for this study are included in the article/Supplementary Materials.

## Supplementary Materials

The following are available online at https://www.mdpi.com/article/10.3390/antiox10071071/s1, Figure S1: (a) Increase in IL-6, TNF-α, NO, and intracellular ROS (measured in percent of control) in RAW 264.7 cells treated with LPS for 24 h. (b) Increase in relative mRNA expressions of IL-1β, IL-6, iNOS, and TNF-α (measured in percent of control) in the RAW 264.7 macrophage cells treated with LPS for 6 h., Figure S2: 1H-NMR (a) and 13C-NMR (b) spectrum of the isolated pyrogallol sample.
