# Supplementary material for: Anti-Oxidant and Anti-Inflammatory Substance Generated Newly in Paeoniae Radix Alba Extract Fermented with Plant-Derived Lactobacillus brevis 174A
Source: Antioxidants (Basel). 2021 Jul 2;10(7):1071. doi: 10.3390/antiox10071071 (PMC8300999; doi:10.3390/antiox10071071)
Supplement: Supplementary file 1 [file antioxidants-10-01071-s001.zip › antioxidants-1292901-supplementary.pdf]

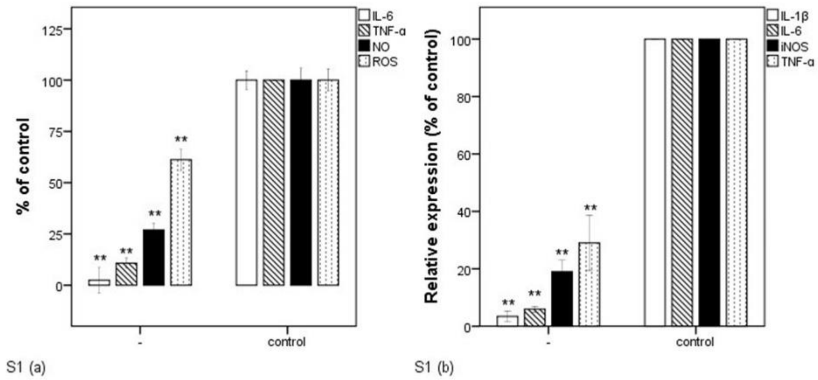

Figure S1. (a) Increase in IL-6, TNF-α, NO, and intracellular ROS (measured in percent of control) in RAW 264.7 cells treated with LPS for 24 h. (b) Increase in relative mRNA expressions of IL-1β, IL-6, iNOS, and TNF-α (measured in percent of control) in the RAW 264.7 macrophage cells treated with LPS for 6 h. Data are expressed as the mean value of at least triplicate experiments. Error bars represent +/- standard deviation. **$p<0.01$ versus control. -: untreated cells; control: cells treated with 1μg/ml LPS.

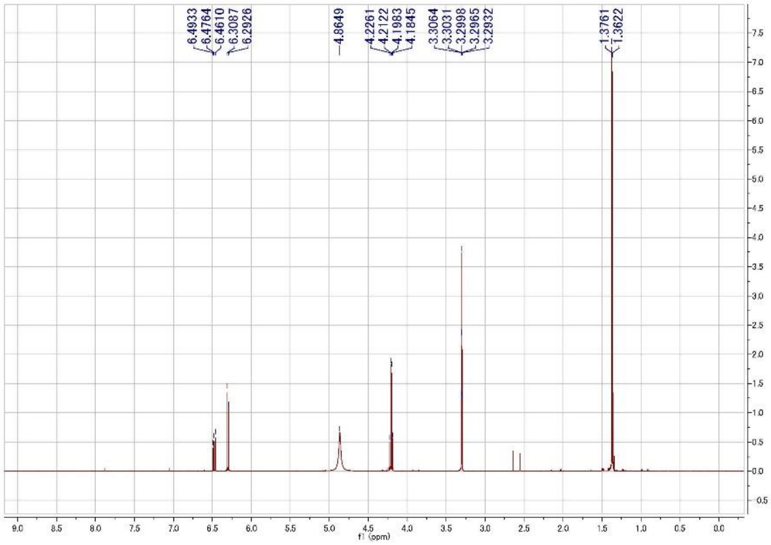

(a) ¹H-NMR spectrum

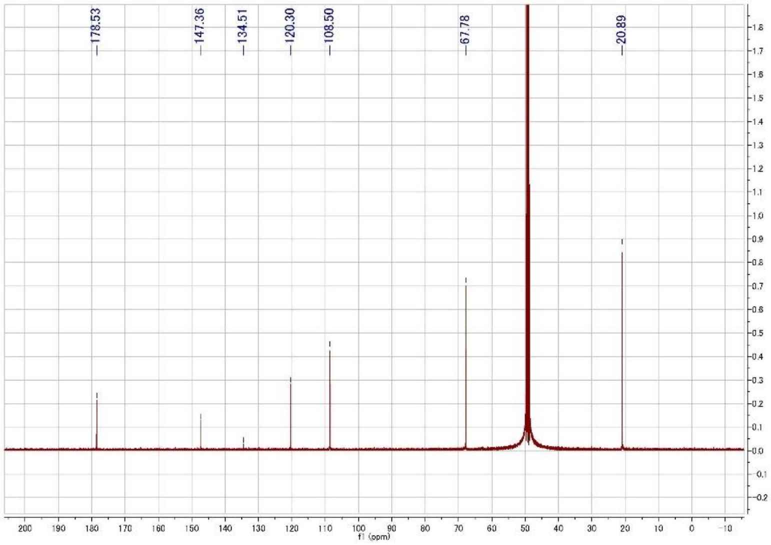

(b) ¹³C-NMR spectrum

Figure S2. NMR spectrum of the isolated pyrogallol sample.
